# Determinants and Strategies for Improving Attendance in Pharmacology: A Mixed-Methods Cross-Sectional Study From a Medical School in Mauritius

**DOI:** 10.7759/cureus.110227

**Published:** 2026-06-04

**Authors:** Anshika Bundhoo, Ritisha Conjobeeharry, Divya Gunnasya, Trishila Anand Bhoma, Sheena Bharati Auckloo, Indrajit Banerjee

**Affiliations:** 1 Pharmacology, Sir Seewoosagur Ramgoolam Medical College, Belle Rive, MUS; 2 General Medicine, Sir Anerood Jugnauth (SAJ) Hospital, Ministry of Health and Wellness, Flacq, MUS

**Keywords:** absenteeism risk, chronic absenteeism, mauritius, medical institutions, medical school students, pharmacology education, pharmacology learning, preclinical medical students, school attendance, undergraduate medical students

## Abstract

Introduction

Absenteeism refers to frequent absence from classes without a valid or justifiable reason. This study aimed to explore medical students’ perceptions of the factors associated with absenteeism in pharmacology classes, identify student-perceived strategies to improve attendance, and examine the associations between demographic variables and students’ perceptions of absenteeism-related factors at Sir Seewoosagur Ramgoolam Medical College, Belle Rive, Mauritius.

Methods

A mixed-method cross-sectional observational study was conducted at Sir Seewoosagur Ramgoolam Medical College, Belle Rive, Mauritius, from October 4 to November 2, 2025. Google Forms (Google Inc., Mountain View, CA, USA) was used to collect the quantitative data. The quantitative data were analyzed using IBM SPSS Statistics for Windows Version 31.0 (IBM Corp., Armonk, NY, USA). The qualitative data were analyzed using NVivo 15 (Windows) software (QSR International, Melbourne, Australia).

Results

During the study period, a total of 144 medical students were enrolled in the pharmacology course, of whom 100 respondents participated in the study, yielding a response rate of 69.44%. Among the respondents, 61 (61%) were female, and 39 (39%) were male. A significant association was identified between semester and perceptions regarding long lectures (> 2 hours) as a contributing factor to absenteeism (p = 0.002) and difficulty of attending classes for six consecutive days (p = 0.046) for factors causing absenteeism in pharmacology. For the factors improving attendance, a relationship was observed between gender and the link to the clinical practice (p = 0.02), with a greater proportion of male students (23, 59.0%) strongly agreeing with this approach compared to female students (26, 42.6%). From the thematic analysis, five themes emerged from the generated codes, which were attendance patterns, academic and teaching influence, social and peer influence, institutional and policy factors, and structural and environmental barriers.

Conclusion

Absenteeism in pharmacology remains a significant and multifactorial issue. This study identifies several contributory factors underlying absenteeism, including long lectures (>1 hour), a preference for self-study, difficulty waking up, six consecutive classes per week, and burnout, which were perceived by students as influencing attendance. The factors that may improve attendance were flexible class schedules, incorporating mixed teaching techniques, providing study breaks before examinations, reducing the duration of lectures, and delaying early morning classes (8.00 am). In addition, making lectures more interactive and linking content to clinical practice are essential and were perceived by students to improve attendance.

## Introduction

Student absenteeism is a pertinent challenge observed across higher academic institutions globally, including medical institutions. Absenteeism refers to frequent nonattendance from scheduled lectures without any reasonable grounds [1]. Despite strict policies on student attendance, high rates of absenteeism persist in medical colleges, reflecting a major global concern [2, 3]. Numerous studies have correlated absenteeism with poorer outcomes, both academically and clinically. For example, skipping key sessions has been linked to compromised clinical competencies such as prescription writing and even challenges in maintaining professionalism [4-7]. From a quality perspective, absenteeism represents a significant waste of educational resources, time, and human potential. It also disrupts the class's overall well-being, creating an unwelcoming teaching-learning environment and negatively affecting even those students who attend regularly [8]. Several studies have identified numerous causes contributing to increased absenteeism among medical students. These include low interest in the subject, ineffective or monotonous teaching methods, an unfavorable learning environment, excessive social activities, part-time job commitments, insomnia, poor health, and strained relationships with faculty. Research has pointed to the easy availability of lecture materials, such as online slides, videos, and audio recordings, as factors that reduce classroom attendance. According to the study conducted by Cook et al., oversleeping was highlighted as the primary reason for student absenteeism [9]. Sir Seewoosagur Ramgoolam Medical College is the first medical college established in 1999 in Mauritius. It is affiliated with the University of Mauritius. The professional course is divided into the first professional (semesters 1-3), second professional (semesters 4-6), final part I (semesters 7-8), and final part II (semesters 9-10) [10]. Pharmacology is taught at the second professional level from semester 4 to 6 [11].

The primary aim of medical education is to produce good clinicians with in-depth knowledge and reasonable clinical skills, which can be achieved with high motivation to attend classes. Pharmacology occupies a pivotal position in the medical curriculum, bridging basic sciences with clinical practice. A thorough understanding of pharmacological principles is essential for the development of safe and rational prescribing practices, which directly influence patient outcomes. 

Despite the observed nature of the problem, there is a dearth of data related to absenteeism in Mauritius. The research was conducted to address this research gap. This mixed-methods cross-sectional study aimed to explore medical students’ perceptions regarding factors associated with absenteeism in pharmacology classes and identify student-perceived strategies to improve attendance at Sir Seewoosagur Ramgoolam Medical College, Belle Rive, Mauritius. The study also examined associations between selected demographic variables and students’ perceptions of absenteeism-related factors. 

This study was presented as a meeting abstract at Health Horizon 2025: 5^th^ International Symposium on Medical Sciences, Mauritius, on September 6, 2025.

## Materials and methods

Study design and the participants

A mixed-method cross-sectional observational study was conducted at Sir Seewoosagur Ramgoolam Medical College, Belle Rive, Mauritius, from October 4 to November 2, 2025. A total of 100 students out of 144 students currently pursuing their second phase of the MBBS course (semesters 4-6) and studying pharmacology were taken into consideration for quantitative data analysis. A total of 12 students were included in the qualitative component of the study. Participants were selected from semesters 4 to 6 and were currently studying pharmacology at the time of the study. The sample comprised an equal distribution of male and female students, with six males and six females. To ensure representation across academic levels, four students were recruited from each semester (semesters 4-6). The participants also represented diverse national backgrounds, including four Mauritian, four Indian, and four South African students. The Strengthening the Reporting of Observational Studies in Epidemiology (STROBE) guidelines were followed for reporting this cross-sectional study [12]. Written informed consent was obtained from all the participants.

Data Collection

Quantitative Domain

A self-designed questionnaire, developed by the investigators, was used for the study. The questionnaire was distributed through an online platform, such as Google Forms (Google Inc., Mountain View, CA, USA), to approximately 100 students from the fourth to the sixth semesters, who were currently studying pharmacology. All personal details of the students remained strictly confidential. 

Qualitative Domain

Open-ended questions were prepared by the researchers for the interview to collect data for the qualitative domain. In-depth interviews were conducted by the researchers (DG and TAB). Each interview lasted approximately 20 minutes on average. The interview data were transcribed using both voice-to-text transcription applications and manual transcription methods to ensure accuracy. Interviews were conducted in a private one-on-one setting under the supervision and guidance of a senior author.

The interviewers were trained in qualitative interviewing techniques and conducted the interviews in a neutral and nonjudgmental manner to minimize interviewer bias. The interviewers had no conflict of interest with the participants. Participants' anonymity and confidentiality were maintained throughout the study process. Reflexivity was maintained through regular discussions among the research team regarding coding decisions, interpretations, and potential preconceptions. Efforts were made to ensure that participants felt comfortable sharing their experiences openly and honestly during the interviews. 

Independent coding of the qualitative data was performed by two researchers (DG and TAB). Intercoder reliability was assessed using Cohen's kappa coefficient, demonstrating substantial agreement between the coders (κ=0.76). Any discrepancies in coding were resolved through discussion and consensus coding under the guidance of the senior author (IB), and the final codes were agreed upon before thematic analysis. 

Methodologicaltriangulation

Methodological triangulation was employed to strengthen the credibility and robustness of this mixed-methods study. The quantitative and qualitative datasets were analyzed independently and subsequently integrated to compare, confirm, and enrich the overall interpretation of the findings. 

Questionnaire* *design

After an extensive review of the literature, a questionnaire was designed by the investigators to meet the objective of the study. The questionnaire consisted of three sections: a. Demographic details, b. Factors causing absenteeism, c. Improvements, and d. Qualitative domain. A 5-point Likert scale was used for the degree of agreement for the factors causing absenteeism and improvements, where 1 = strongly disagree, 2 = disagree, 3 = neutral, 4 = agree, and 5 = strongly agree (Appendix A). 

Questionnairevalidityandreliability

The questionnaire was validated by five subject experts for construct, content, and criterion validity. The average congruence percentage was 90%, indicating strong content validity. Cronbach’s alpha was used to assess the internal consistency reliability of the questionnaire (Quantitative domain). A pilot study was done among 10 students (semesters 4-6). Cronbach’s alpha value was found to be 0.821, indicating good reliability and acceptable internal consistency. For the quantitative method, a set of open-ended questions was prepared for the interview. 

Face validity

A pilot study was conducted among 10 students from all three semesters for readability and understanding of the questionnaire. 

*​​​*Inclusion* *criteria

A total of 100 students out of 144 students currently pursuing the second phase of the MBBS course from semesters 4-6 and studying pharmacology at Sir Seewoosagur Ramgoolam Medical College, were included for the quantitative domain. A total of 12 students were included for qualitative data analysis from semesters 4-6 and were labelled as (A1-A12). Participants were selected from semesters 4 to 6 and were currently studying pharmacology at the time of the study. The sample comprised an equal distribution of male and female students, with six males an tvd six females. To ensure representation across academic levels, four students were recruited from each semester (semesters 4-6). The participants also represented diverse national backgrounds, including four Mauritian, four Indian, and four South African students.

Exclusion criteria 

Students from semesters 1-3 were excluded as they were not exposed to pharmacology. Furthermore, semesters 7-10 were also excluded from the study to avoid recall bias. Incomplete questionnaires and students who did not give consent were excluded from this study.

Ethical approval

Prior to the data collection, ethical approval was obtained from the Institutional Research and Ethics Committee of Sir Seewoosagur Ramgoolam Medical College on October 3, 2025 (approval code: SSRMC/IERB/2025/008). The research was conducted in accordance with the Declaration of Helsinki.

Sample size calculation

The sample size was determined by using the following formula [13].

$z^2 p(1-p)/d^2$ 

Assuming a 95% confidence level (Z = 1.96), a margin of error of 5% (d = 0.05), and an estimate of population proportion (p = 0.5), to ensure maximum variability. The calculated sample size was adjusted considering the finite population of 144 eligible participants.

A total population sampling technique was applied by inviting all eligible participants in the study. Of these 100 students who completed the questionnaire, were included yielded a response rate of 69.44%.

For the qualitative component, the data were collected according to the guidelines by Glaser et al, and the interviews were conducted until data saturation was reached, that is, no new codes/ nodes were generated from the interviews [14].

Data management and statistical analysis

Quantitative Data

The quantitative data were analyzed using IBM SPSS Statistics for Windows Version 31.0 (IBM Corp., Armonk, NY, USA). Chi-square test was used to assess associations between categorical variables. When the assumptions for chi-square analysis were violated, particularly when expected cell frequencies were less than five in contingency tables, the Fisher-Freeman-Halton exact test was used for larger R X C (rows x columns) contingency tables. A p-value of less than 0.05 was regarded as statistically significant. 

Before analysis, the dataset underwent a data cleaning process that included identifying and addressing missing values through appropriate imputation methods, detecting and managing outliers using standard deviation-based criteria, and correcting inconsistencies through systematic data validation procedures. 

Qualitative Data

The qualitative data were analysed using NVivo 15 (Windows) software (QSR International, Melbourne, Australia). Braun and Clarke’s six-step thematic analysis method was used for inductive thematic phenomenological analysis [15].

Thematic analysis was conducted using a six-phase approach consisting of the following steps: i. familiarization with the data, where the authors read the data and transcripts and noted initial ideas and observations; ii. generation of the initial codes through systematic identification and labelling was performed; iii. themes were identified via the collation and grouping of the specific nodes and data generated; iv. themes, in relation to the extracted codes, were reviewed and further refined; v. defining and naming the specific identified themes were done; and vi. through specific examples, the final analysis was done, and the findings of the study were reported.

## Results

Quantitative domain

During the study period, a total of 144 medical students were enrolled in the pharmacology course, of whom 100 participated, yielding a response rate of 100 (69.44%). Among the respondents, 61 (61%) were female, and 39 (39%) were male. As shown in Table 1, the majority of participants were from semester 6 (39%), followed by semester 5 (33%) and semester 4 (28%). With respect to nationality, more than half of the students were Mauritian (51, 51%), while 30(30%) were Indian, 18 (18%) were South African, and one respondent (1%) was from Rodrigues.

**Table 1:** Demographic details of the participants ^x^p >0.05 (statistically insignificant); chi-square test was used to calculate p-values. The Fisher–Freeman–Halton exact test was used to calculate p-values (cells < 5 counts).

Demographic profile (n=100)		Female n (%)	Male n (%)	Total n (%)	Chi-square (χ² value)/Fisher–Freeman–Halton exact test	P-value
Semesters	4^th^	18(29.5)	10(25.6)	28(28)	3.41	0.182^X^
	5^th^	16(26.2)	17(43.6)	33(33)		
	6^th^	27(44.3)	12(30.8)	39(39)		
Nationality	Mauritian	34(55.7)	17(43.6)	51(51)	Fisher–Freeman–Halton exact test	0.393^X^
	Indian	16 26.2)	14(35.9)	30(30)		
	South African	11(18.0)	7(17.9)	18(18)		
	Rodrigues	0(0)	1(100)	1(1)		
Current living arrangement	Day scholar	52(86.7)	33(84.6)	86(86)	0.082	0.775^X^
	Hostellers	8(13.3)	6(15.4)	14(14)		
Commute to college	Bus	25(41.7)	13(33.3)	38(38)	0.83	0.66^X^
	Car	27(45.0)	19(48.7)	48(48)		
	Walk	8(13.3)	7(17.9)	14(14)		

A statistically significant association was observed between the semester and the reported difficulty of attending classes for six consecutive days (p = 0.046). Sixth-semester students exhibited the highest level of agreement, with 27(69.2%) strongly agreeing, whereas fifth-semester students reported comparatively lower levels of difficulty. No statistically significant relationship was found between nationality and difficulty waking up for early morning lectures (p = 0.058). Nonetheless, a greater proportion of Indian (16, 53.3%) and South African (9, 50.0%) students strongly agreed that they experienced difficulty waking up for early morning sessions. A significant association was also identified between semester and perceptions regarding long lectures (> 1 hour) as a contributing factor to absenteeism (p = 0.002). Specifically, 11 (33.3%) of fifth-semester students strongly agreed, and 0 (0.0%) strongly disagreed that such lectures were a major factor influencing absenteeism (Table 2).

**Table 2:** Associations between factors causing absenteeism and semester/gender/nationality ** p<0.05 (statistically significant) ^x^p >0.05 (statistically insignificant). The Fisher–Freeman–Halton exact test was used to calculate p-values (cells < 5 counts).

Factors causing absenteeism		Strongly Agree	Agree	Neutral	Disagree	Strongly Disagree	P-value
Semester	4^th^	2(7.1)	6(21.4)	16(57.1)	1(3.6)	3(10.7)	0.002**
	5^th^	11(33.3)	11(33.3)	7(21.2)	4(12.1)	0(0.0)	
	6^th^	6(15.4)	8(20.5)	10(25.6)	11(28.2)	4(10.3)	
Gender	Female	11(18.0)	14(23.0)	21(34.4)	10(16.4)	5(5(8.2)	0.942^X^
	Male	8(20.5)	11(28.2)	12(30.8)	6(15.4)	2(5.1)	
Nationality	Indian	7(23.3)	7(23.3)	10(33.3)	3(10.0)	3(10.0)	0.467^X^
	Mauritian	6(11.8)	12(23.5)	18(35.3)	12(23.5)	3(5.9)	
	South African	5(27.8)	6(33.3)	5(27.8)	1(5.6)	1(5.6)	
	Rodrigues	1(100)	0 (0)	0 (0)	0 (0)	0 (0)	
Semester	4^th^	2(7.1)	7(25.0)	11(39.3)	3(10.7)	5(17.9)	0.175^X^
	5^th^	11(33.3)	4(12.1)	10(30.3)	5(15.2)	3(9.1)	
	6^th^	5(12.8)	7(17.9)	11(28.2)	9(23.1)	7(17.9)	
Gender	Female	10(16.4)	10(16.4)	16(26.2)	12(19.7)	13(21.3)	0.142^X^
	Male	8(20.5)	8(20.5)	16(41.0)	5(12.8)	2(5.1)	
Nationality	Indian	9(30.0)	4(13.3)	6(20.0)	6(20.0)	5(16.7)	0.243^X^
	Mauritian	4(7.8)	9(17.6)	21(41.2)	9(17.6)	8(15.7)	
	South African	4(22.2)	5(27.8)	5(27.8)	2(11.1)	2(11.1)	
	Rodrigues	1(100)	0 (0)	0 (0)	0 (0)	0 (0)	
Semester	4^th^	6(21.4)	10(35.7)	6(21.4)	2(7.1)	4(14.3)	0.312^X^
	5^th^	12(36.4)	6(18.2)	9(27.3)	2(6.1)	4(12.1)	
	6^th^	19(48.7)	6(15.4)	11(28.2)	1(2.6)	2(5.1)	
Gender	Female	23(37.7)	15(24.6)	14(23.0)	2(3.3)	7(11.5)	0.676^X^
	Male	14(35.9)	7(17.9)	12(30.8)	3(7.7)	3(7.7)	
Nationality	Indian	11(36.7)	5(16.7)	9(30.0)	2(6.7)	3(10.0)	0.721^X^
	Mauritian	15(29.4)	13(25.5)	13(25.5)	3(5.9)	7(13.7)	
	South African	10(55.6)	4(22.2)	4(22.2)	0 (0)	0 (0)	
	Rodrigues	1(100)	0 (0)	0 (0)	0 (0)	0 (0)	
Semester	4^th^	9(32.1)	5(17.9)	5(17.9)	4(14.3)	5(17.9)	0.685^X^
	5^th^	8(24.2)	4(12.1)	7(21.2)	7(21.2)	7(21.2)	
	6^th^	16(41.0)	8(20.5)	7(17.9)	5(12.8)	3(7.7)	
Gender	Female	17(27.9)	9(14.8)	13(21.3)	12(19.7)	10(16.4)	0.452^X^
	Male	16(41.0)	8(20.5)	6(15.4)	4(10.3)	5(12.8)	
Nationality	Indian	16(53.3)	4(13.3)	5(16.7)	2(6.7)	3(10.0)	0.058^X^
	Mauritian	7(13.7)	10(19.6)	13(25.5)	11(21.6)	10(19.6)	
	South African	9(50.0)	3(16.7)	1(5.6)	3(16.7)	2(11.1)	
	Rodrigues	1(100)	0 (0)	0 (0)	0 (0)	0 (0)	
Semester	4^th^	4(14.3)	9(32.1)	12(42.9)	2(7.1)	1(3.6)	0.416^X^
	5^th^	9(27.3)	11(33.3)	7(21.2)	4(12.1)	2(6.1)	
	6^th^	15(38.5)	12(30.8)	8(20.5)	3(7.7)	1(2.6)	
Gender	Female	18(29.5)	19(31.1)	18(29.5)	4(6.6)	2(3.3)	0.778^X^
	Male	10(25.6)	13(33.3)	9(23.1)	5(12.8)	2(5.1)	
Nationality	Indian	9(30.0)	8(26.7)	7(23.3)	4(13.3)	2(6.7)	0.534^X^
	Mauritian	11(21.6)	20(39.2)	16(31.4)	2(3.9)	2(3.9)	
	South African	7(38.9)	4(22.2)	4(22.2)	3(16.7)	0 (0)	
	Rodrigues	1(100)	0 (0)	0 (0)	0 (0)	0 (0)	
Semester	4^th^	17(60.7)	3(10.7)	6(21.4)	0 (0)	2(7.1)	0.046**
	5^th^	13(39.4)	7(21.2)	4(12.1)	6(18.2)	3(9.1)	
	6^th^	27(69.2)	3(7.7)	6(15.4)	1(2.6)	2(5.1)	
Gender	Female	35(57.4)	9(14.8)	9(14.8)	3(4.9)	5(8.2)	0.77^X^
	Male	22(56.4)	4(10.3)	7(17.9)	4(10.3)	2(5.1)	
Nationality	Indian	15(50.0)	5(16.7)	5(16.7)	3(10.0)	2(6.7)	0.99^X^
	Mauritian	29(56.9)	6(11.8)	8(15.7)	4(7.8)	4(7.8)	
	South African	12(66.7)	2(11.1)	3(16.7)	0 (0)	1(5.6)	
	Rodrigues	1(100)	0 (0)	0 (0)	0 (0)	0 (0)	
Semester	4^th^	7(25.0)	5(17.9)	6(21.4)	3(10.7)	7(25.0)	0.852^X^
	5^th^	7(21.2)	7(21.2)	8(24.2)	4(12.1)	7(21.2)	
	6^th^	13(33.3)	11(28.2)	5(12.8)	4(10.3)	6(15.4)	
Gender	Female	16(26.2)	14(23.0)	13(21.3)	7(11.5)	11(18.0)	0.937^X^
	Male	11(28.2)	9(23.1)	6(15.4)	4(10.3)	9(23.1)	
Nationality	Indian	11(36.7)	8(26.7)	5(16.7)	1(3.3)	5(16.7)	0.393^X^
	Mauritian	10(19.6)	9(17.6)	11(21.6)	7(13.7)	14(27.5)	
	South African	5(27.8)	6(33.3)	3(16.7)	3(16.7)	1(5.6)	
	Rodrigues	1(100)	0 (0)	0 (0)	0 (0)	0 (0)	
Semester	4^th^	3(10.7)	1(3.6)	9(32.1)	7(32.1)	8(28.6)	0.251^X^
	5^th^	5(15.2)	2(6.1)	7(21.2)	7(21.2)	12(36.4)	
	6^th^	12(30.8)	5(12.8)	10(25.6)	5(12.8)	7(17.9)	
Gender	Female	10(16.4)	6(9.8)	19(31.1)	10(16.4)	16(26.2)	0.425^X^
	Male	10(25.6)	2(5.1)	7(17.9)	9(23.1)	11(28.2)	
Nationality	Indian	6(20.0)	2(6.7)	9(30.0)	5(16.7)	8(26.7)	0.099^X^
	Mauritian	10(19.6)	2(3.9)	17(33.3)	11(21.6)	11(21.6)	
	South African	3(16.7)	4(22.2)	0 (0)	3(16.7)	8(44.4)	
	Rodrigues	1(100)	0(0)	0(0)	0(0)	0(0)	

Table 3 depicts the association between gender and factors perceived to improve attendance. A statistically significant relationship was observed between gender and the link to the clinical practice (p= 0.02), with a greater proportion of male students (23, 59.0%) strongly agreeing with this approach compared to female students (26, 42.6%). Students in semester 4 (15, 53.6%) were most supportive of this measure, followed by those in semester 5 (17, 51.5%) and Semester 6 (17, 43.6%). No statistically significant association was observed between nationality and the perception that early morning lectures should be delayed (p > 0.05). However, South African students (11, 61.1%) were most in favor of this adjustment, followed by Indian students (19, 63.3%) and Mauritian students (16, 31.4%)

**Table 3:** Association between factors improving attendance and semester/gender/nationality ** p<0.05 (statistically significant) ^x^p >0.05 (statistically insignificant). The Fisher–Freeman–Halton exact test was used to calculate p-values (cells < 5 counts)

Factors improving attendance		Strongly Agree	Agree	Neutral	Disagree	Strongly Disagree	P-value
Semester	4^th^	6(21.4)	2(7.1)	17(60.7)	3(10.7)	0(0.0)	0.123^X^
	5^th^	10(30.3)	2(6.1)	15(45.5)	3(9.1)	3(9.1)	
	6^th^	17(43.6)	6(15.4)	11(28.2)	3(7.7)	2(5.1)	
Gender	Female	17(27.9)	4(6.6)	32(52.5)	5(8.2)	3(4.9)	0.942^X^
	Male	16(41.0)	6(15.4)	11(28.2)	4(10.3)	2(5.1)	
Nationality	Indian	7(23.3)	3(10.0)	14(46.7)	4(13.3)	2(6.7)	0.467^X^
	Mauritian	22(43.1)	2(3.9)	24(47.1)	2(3.9)	1(2.0)	
	South African	3(16.7)	5(27.8)	5(27.8)	3(16.7)	2(11.1)	
	Rodrigues	1(100.0)	0 (0.0)	0 (0.0)	0 (0.0)	0 (0.0)	
Semester	4^th^	15(53.6)	4(14.3)	5(17.9)	1(3.6)	3(10.7)	0.088^X^
	5^th^	18(54.5)	5(15.2)	5(15.2)	2(6.1)	3(9.1).7	
	6^th^	23(59.0)	9(23.1)	5(12.8)	1(2.6)	1(2.6)	
Gender	Female	36(59.0)	11(18.0)	8(13.1)	1(1.6)	5(8.2)	0.053^X^
	Male	20(51.3)	7(17.9)	7(17.9)	3(7.7)	2(5.1)	
Nationality	Indian	18(60.0)	3(10.0)	6(20.0)	2(6.7)	1(3.3)	0.092^X^
	Mauritian	27(52.9)	12(23.5)	7(13.7)	1(2.0)	4(7.8)	
	South African	10(55.6)	3(16.7)	2(11.1)	1(5.6)	2(11.1)	
	Rodrigues	0 (0.0)	1(100.0)	0 (0.0)	0 (0.0)	0 (0.0)	
Semester	4^th^	6(21.4)	7(25.0)	12(42.9)	2(7.1)	1(3.6)	0.174^X^
	5^th^	12(36.4)	7(21.2)	12(36.4)	1(3.0)	1(3.0)	
	6^th^	11(28.2)	4(10.3)	12(30.8)	8(20.5)	4(10.3)	
Gender	Female	15(24.6)	7(11.5)	26(42.6)	8(13.1)	5(8.2)	0.073^X^
	Male	14(35.9)	11(28.2)	10(25.6)	3(7.7)	1(2.6)	
Nationality	Indian	8(26.7)	6(20.0)	14(46.7)	1(3.3)	1(3.3)	0.659^X^
	Mauritian	14(27.5)	8(15.7)	19(37.3)	7(13.7)	3(5.9)	
	South African	6(33.3)	4(22.2)	3(16.7)	3(16.7)	2(11.1)	
	Rodrigues	1 (100.0)	0 (0.0)	0(0.0)	0 (0.0)	0 (0.0)	
Semester	4^th^	15(53.6)	9(32.1)	3(10.7)	1(3.6)	0 (0.0)	0.062^X^
	5^th^	17(51.5)	5(15.2)	9(27.3)	1(3.0)	1(3.0)	
	6^th^	17(43.6)	9(23.1)	9(23.1)	2(5.1)	2(5.1)	
Gender	Female	26(42.6)	15(24.6)	15(24.6)	4(6.6)	1(1.6)	0.020**
	Male	23(59.0)	8(20.5)	6(15.4)	0(0.0)	2(5.1)	
Nationality	Indian	13(43.3)	7(23.3)	7(23.3)	1(3.3)	2(6.7)	0.986^X^
	Mauritian	25(49.0)	12(23.5)	11(21.6)	2(3.9)	1(2.0)	
	South African	10(55.6)	4(22.2)	3(16.7)	1(5.6)	0 (0.0)	
	Rodrigues	1(100.0)	0 (0.0)	0 (0.0)	0 (0.0)	0 (0.0)	
Semester	4^th^	21(75.0)	6(21.4)	1(3.6)	0 (0.0)	0 (0.0)	0.058^X^
	5^th^	27(81.8)	2(6.1)	3(9.1)	1(3.0)	0 (0.0)	
	6^th^	37(94.9)	1(2.6)	0 (0.0)	0 (0.0)	1(2.6)	
Gender	Female	53(86.9)	4(6.6)	3(4.9)	0 (0)	1(1.6)	0.458^X^
	Male	32(82.1)	5(12.8)	1(2.6)	1(2.6)	0 (0.0)	
Nationality	Indian	25(83.3)	2(6.7)	2(6.7)	0 (0.0)	1(3.3)	0.926^X^
	Mauritian	42(82.4)	6(11.8)	2(3.9)	1(2.0)	0 (0.0)	
	South African	17(94.4)	1(5.6)	0 (0.0)	0 (0.0)	0 (0.0)	
	Rodrigues	1(100.0)	0 (0.0)	0 (0.0)	0 (0.0)	0 (0.0)	
Semester	4^th^	14(50.0)	3(10.7)	3(10.7)	6(21.4)	2(7.1)	0.231^X^
	5^th^	14 (42.4)	5(15.2)	9(27.3)	2(6.1)	3(9.1)	
	6^th^	19(48.7)	10(25.6)	6(15.4)	2(5.1)	2(5.1)	
Gender	Female	27(44.3)	12(19.7)	12(19.7)	6(9.8)	4(6.6)	0.941^X^
	Male	20(51.3)	6(15.4)	6(15.4)	4(10.3)	3(7.7)	
Nationality	Indian	19(63.3)	4(13.3)	5(16.7)	0 (0.0)	2(6.7)	0.054^X^
	Mauritian	16(31.4)	11(21.6)	12(23.5)	7(13.7)	5(9.8)	
	South African	11(61.1)	3(16.7)	1(5.6)	3(16.7)	0 (0.0)	
	Rodrigues	1(100.0)	0 (0.0)	0 (0.0)	0 (0.0)	0 (0.0)	
Semester	4^th^	10(35.7)	11(39.3)	3(10.7)	2(7.1)	2(7.1)	0.393^X^
	5^th^	23(69.7)	7(21.2)	1(3.0)	1(3.0)	1(3.0)	
	6^th^	17(43.6)	14(35.9)	4(10.3)	2(5.1)	2(5.1)	
Gender	Female	28(45.9)	19(31.1)	6(9.8)	4(6.6)	4(6.6)	0.603^X^
	Male	22(56.4)	13(33.3)	2(5.1)	1(2.6)	1(2.6)	
Nationality	Indian	19(63.3)	8(26.7)	0 (0.0)	1(3.3)	2(6.7)	0.419^X^
	Mauritian	20(39.2)	20(39.2)	7(13.7)	3(5.9)	1(2.0)	
	South African	10(55.6)	4(22.2)	1(5.6)	1(5.6)	2(11.1)	
	Rodrigues	0(0.0)	0(0.0)	0 (0.0)	1(100.0)	0(0.0)	

**Figure 1:**
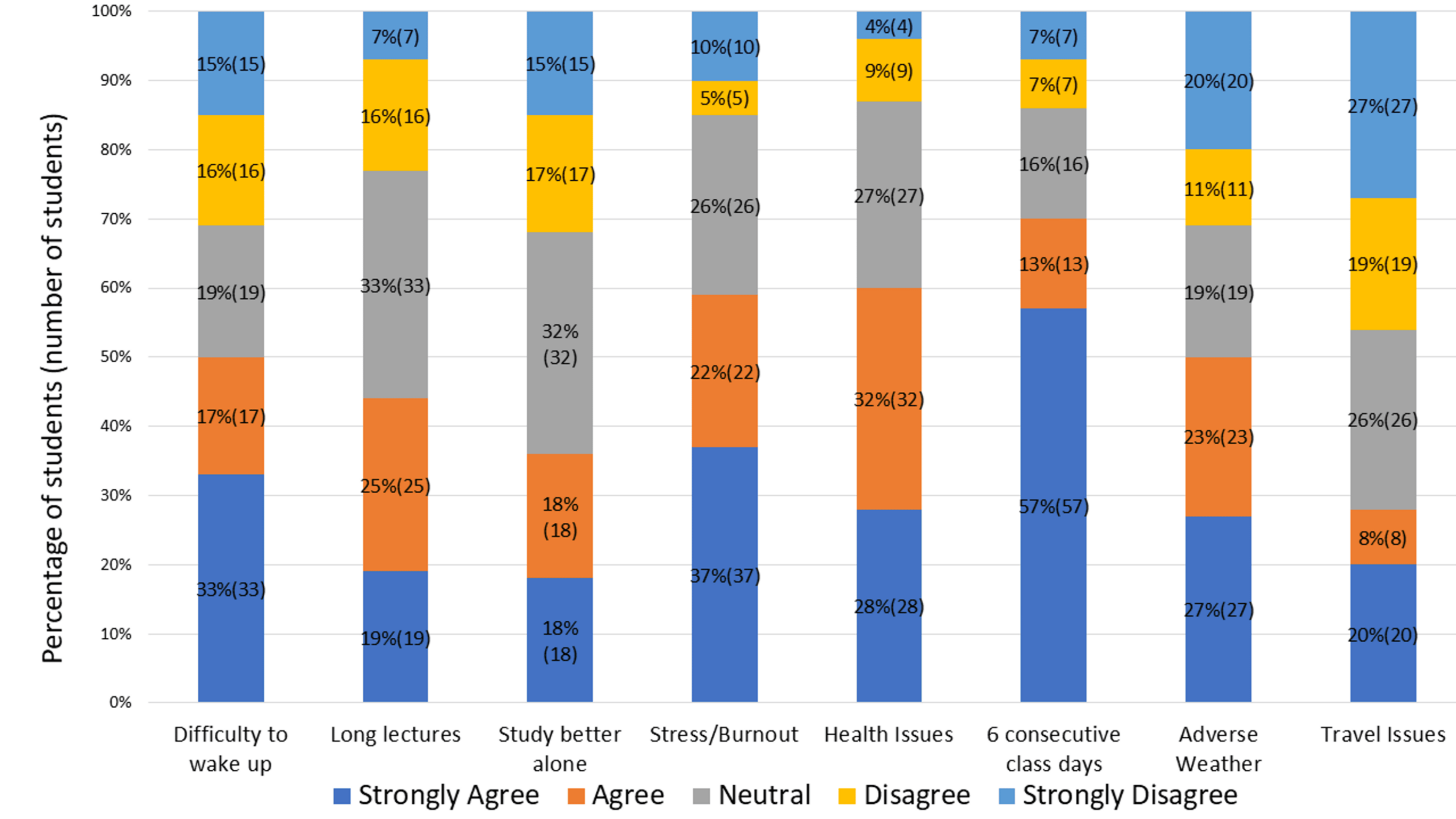
Causes of absenteeism The data have been represented as percentages (%) and total counts (n).

Figure 1 demonstrates the various factors contributing to absenteeism in pharmacology. Among the students, 33% strongly agreed that difficulty waking up early for morning lectures is a factor causing absenteeism, as shown by the first bar. However, the second bar illustrates that 33% (33) of students are neutral about long lectures (> 1 hour) contributing to their absenteeism. The third bar shows a more divided opinion, whereby 32% (32) of students are neutral about studying better on their own. The fourth bar depicts stress and burnout among students as the second leading factor for absenteeism, with 37% strongly agreeing with this. Of the students, 32% agreed that health issues contributed to their absenteeism, as depicted by the fifth bar. The sixth bar substantiates six consecutive lecture days per week as the leading factor for absenteeism, with a majority of 57% (57) of students strongly agreeing with the same. The seventh bar clearly shows that 27% (27) of students strongly agreed that adverse weather contributed to their absenteeism. Finally, the eighth bar demonstrates that travel issues were not a major contributing factor to absenteeism, as 27% (27) of students strongly disagreed, while 26% (26) were neutral about it.

**Figure 2:**
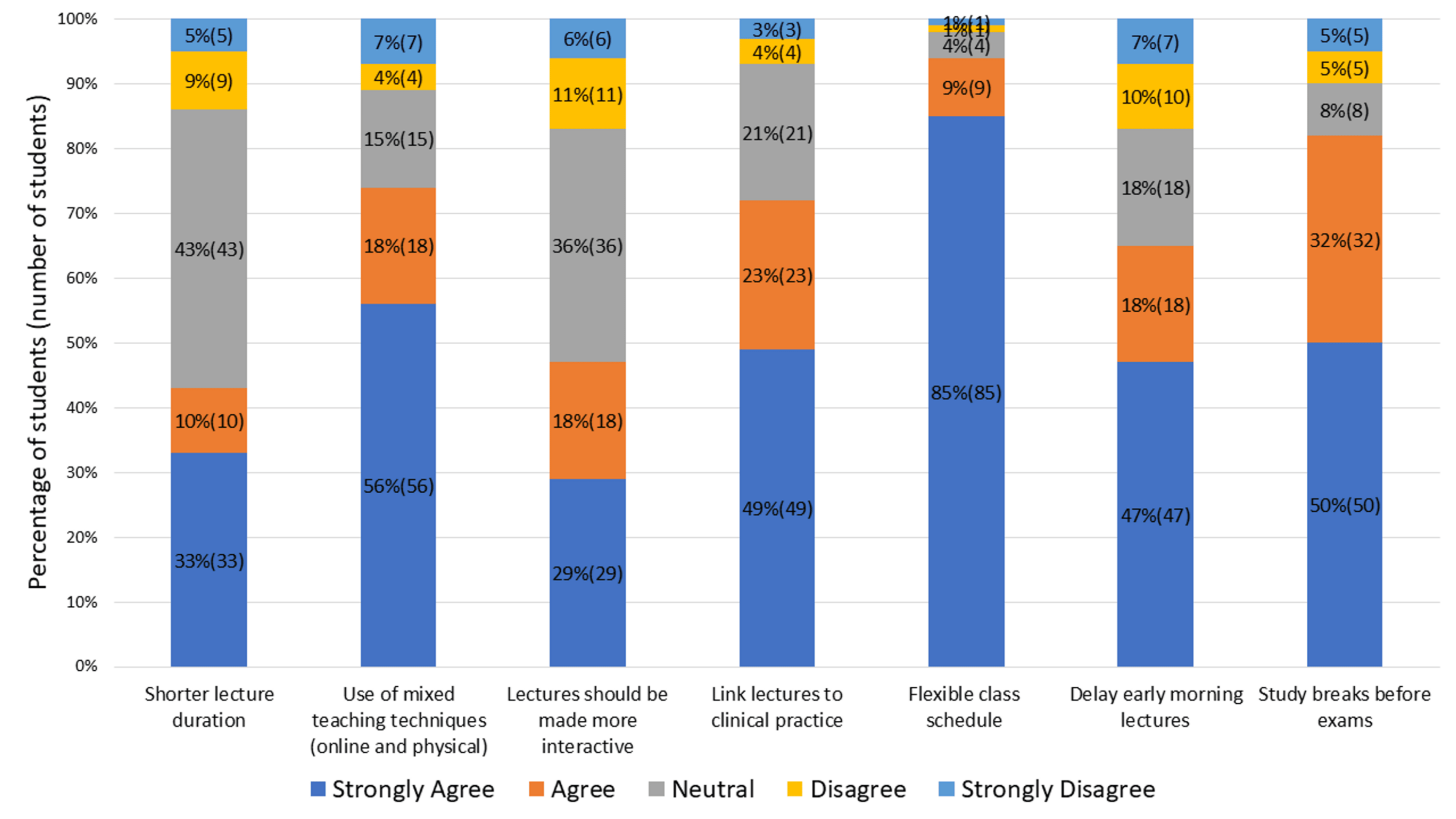
Strategies for improvements of attendance in pharmacology. The data have been represented as percentages (%) and total counts (n).

Figure 2 depicts the various measures that may improve attendance in pharmacology. For reducing the duration of lectures, the majority of students (43, 43%) remained neutral, as shown by the first bar. The use of mixed teaching methods was highly favoured by students, with 56% (56) strongly agreeing, as can be seen in bar 2. The third bar shows that most students (36, 36%) stayed neutral regarding lectures being made more interactive. Linking lectures to clinical practice, as evidenced by the fourth bar, showed 49% (49) of the students strongly agreed to this. Having more flexible class schedules was the most popularly accepted improvement, with 85% (85) of students strongly agreeing to this factor, as demonstrated by the fifth bar. Positive responses were also seen with delaying the morning lectures, with 47% (47) of students strongly agreeing to this, as shown by the sixth bar. Finally, as shown by bar 7, half of the study population (50, 50%) strongly agreed that there should be study breaks before exams.

Qualitative domain

Twelve students (A1-12) were interviewed as part of the qualitative component of the study. Of these, six were male, and six were female, four students from each semester (semesters 4-6), four Mauritian, four Indian, and four were South African participants. At the time of the interviews, all respondents were in their second year of the MBBS program and undertaking pharmacology.

An inductive thematic analysis was conducted, resulting in the generation of five overarching themes: attendance patterns, academic and teaching influences, social and peer influences, institutional and policy factors, and structural and environmental barriers. Each theme encompassed several categories, with each category comprising related codes derived from the data. The findings are summarized in Table 4.

**Table 4:** Thematic phenomenological analysis

Themes	Categories	Codes/Nodes
Attendance patterns	Consistency of attendance	Regular
	Selective participation	Selective attendance
	Routine formation	Routine maintenance
	Learning-driven attendance	Knowledge gain
Academic and teaching influence	Teaching style	Lack of engagement
		Proactive teachers
	Learning methodology	Clinical application
		Self-learning
	Cognitive factors	Burn out
Peer influence	Peer dynamics	Peers influence
Institutional and policy factors	Systemic limitations	Systemic inflexibility
Structural barriers	Scheduling issues	Timing and schedule issues

The codes/nodes are as follows: regular, burnout, selective attendance, clinical application, peer influence, exam eligibility, self-learning, proactive teachers, lack of engagement, system inflexibility, timing and schedule issues, knowledge gain, and routine maintenance. All generated codes/nodes have been organized in Table 5.

**Table 5:** Code/nodes book framework

Codes/Nodes	Description	Narratives
Regular	The consistency with which students attend pharmacology classes.	“I would say I did not skip as many classes as I have an attendance of above 90%” (A2). “I am pretty regular, I usually attend my classes” (A11).
Burnout	Mental overload can lead to skipping classes.	“Burnout affects me a lot. When I’m saturated, I cannot gain more knowledge in my brain, I usually skip class” (A11).
Selective attendance	Students usually prefer to attend classes that are more interactive.	“I am likely to attend interactive classes and not the ones where you only copy the notes” (A1). “If it is interactive, I am more likely to attend” (A7).
Clinical application	Teaching connected to real-life applications and case-based learning encourages students to attend classes.	“If they bring in hospital situations and cases, and when it is a more interactive class” (A5). “Case-based and its relevance to clinical application” (A2).
Peers influence	Students tend to skip classes when their friends are not attending.	“If none of my friends are coming to class, I will obviously bunk”(A8). “If they aren’t coming, I prefer not to come” (A10).
Exams eligibility	Students are motivated to attend classes because of 90% attendance requirement to sit for exams.	“The fact that I need attendance to write” (A6). I usually track my attendance, I try to keep it above the criteria they gave, like 75%, 80% ”(A5).
Self learning	Students want a different approach to learning because they feel like they can read from the book on their own.	“They copy the exact thing that’s in the book, so we could have stayed home and studied” (A4). “To be honest, it’s a lot of self-learning at this point” (A9).
Proactive teachers	Teachers who actively explain concepts motivate attendance.	“Passionate teachers” (A5). “So teachers have different methods of teaching, and some of them, for some teachers, I understand better, so I ensure that I attend those teachers' classes more, and I try to be present in those teachers' classes” (A12).
Lack of engagement	Students sometimes find lectures boring and find it difficult to follow due to a monotonous teaching style.	“Teachers just read off the slides” (A3).
System inflexibility	Illness, medical emergencies, and family emergencies are still counted as absences.	“When I’m sick, it counts as absence” (A1).
Timing and schedule Issues	Students are more likely to bunk early morning classes, long lecture duration, or when there are no afternoon lectures.	“I’m more likely to be absent for the early morning classes” (A6).
Knowledge gain	Students attend when they feel learning value.	“I learn important things” (A2)“. Yes, I would because all the important points are given by the teacher, and it is like good for the exams, and also if you want to read the book is very bulky” (A4).
Routine maintenance	Attendance helps maintain daily structure.	“It brings a routine” (A8).

Table 6 shows the relationship between qualitative and quantitative data. A considerable proportion of students have agreed to academic-related issues. Scheduling-related factors, such as six consecutive days of lectures, have been highly agreed upon; this shows that academic workload and fatigue significantly influence attendance. Other important contributors are health issues, stress, and burnout, thus reflecting the impact of physical and psychological well-being on student participation. Other compounding factors would be external factors such as adverse weather conditions and travel-related issues. Another relatively high agreement point is difficulty waking, showing the influence of lifestyle and sleep scheduling issues. All in all, the findings show an interplay of academic, structural, personal well-being, and environmental constraints.

**Table 6:** Relationship between qualitative and quantitative data.

Quantitative factor (% agree/p-value)	Qualitative theme	Relationship between the two
Classes for six consecutive days a week (69.2% of sixth-semester students; p=0.046)	Institutional and policy factors: rigid schedule	Both data sets support timetable issues as a cause for absenteeism
Stress/Burnout	Academic and teaching influence: cognitive factors. “Burnout affects me a lot…I usually skip class.”	The interviews support the quantitative data findings.
Self-study (36% of students agreed that they would rather study on their own)	Academic and teaching influence: self-learning. Students admitted that they already do a lot of self-learning.	While quantitative data do not reflect self-study as a major cause for absenteeism, it was agreed upon by most interviewees.
Difficulty in waking up (agreed upon by a majority of Indian and South African students) and delaying early morning classes as an improvement	Structural Barriers: timing and schedule issues	These two data sets strongly agree with each other and place early morning classes as a major issue.
Clinical application (p=0.02 for correlation with gender)	Academic and teaching Influence: learning methodology	Students cited that they would be more likely to attend classes if they were related to clinical practice, and the same premise was established in the quantitative domain.

## Discussion

Quantitative domain

Student absenteeism is a prevalent challenge for universities globally. Various studies have shown that absenteeism negatively affects the capabilities of the medical student as a future medical professional. Pharmacology, as a pillar of medical training, plays an indispensable role in the development of skills, both academically and clinically, of a competent doctor. Nevertheless, the growing prevalence of absenteeism in this subject represents a significant but underreported issue that may pose a substantial risk to the future of healthcare worldwide. Therefore, the primary aim of conducting this study was to identify the factors contributing to medical students’ absenteeism and attendance in pharmacology. Secondarily, this research also studies how the patterns of absenteeism differ by semester, gender, and nationality.

The findings of this study have been divided into three categories: factors contributing to absenteeism, factors leading to better attendance, and factors not significantly associated with absenteeism.

Factors Contributing to Absenteeism

Eight factors were taken into consideration for precipitating absenteeism, and they were long lectures (> 1 hour), self-study, stress and burnout, difficulty waking up, health issues, six consecutive classes a week, weather, and travel. The one that was the most statistically significant (p=0.002) was reported to be long lectures with the highest support among the fifth semester students (11, 33.3%). This demonstrated that students strongly believed that having to sit in lectures for extended periods of time would lead them to be more absent from the lectures. This result was also highlighted in a study by Rodriguez-Lara et al., which found “students identified that pedagogical deficiencies such as monotonous and ‘boring lectures’ were negatively affecting attendance” [16].

Six consecutive classes a week were also found to contribute to absenteeism (p=0.046). Students, especially among semester 6 (69.2%), reported that having six classes was exhausting, and this made them want to skip pharmacology classes, which was also cited as a primary reason in the study of Din et al. [17]. A parallel pattern observed among the exam-going semester 6 students was that stress and burnout negatively affect their attendance in lectures (48.7%). This was also reported in the study by Narayankar et al., which established the “top reason for absenteeism being physical exhaustion” [18].

The preference to self-study was another cited reason for absenteeism in pharmacology lectures, as students stated that similar content could be found in books. The Semester 5 students strongly correlated to this factor, among whom 33.3% strongly agreed that self-study was the reason for their absenteeism. Similar patterns were found in a study by Rehman et al., where students “undervalued lectures because they perceived self-study as more efficient than dry, teacher-dominated sessions” [19]. A notably high percentage (53.3%) of students, especially those of Indian nationality, expressed strong agreement that difficulty waking up is their reason for not being present for lectures. “Reported factors included getting up late," which was cited as a reason for absenteeism in Nagappan et al. [20].

Factors Leading to Better Attendance

In terms of factors improving attendance of students in pharmacology lectures, a myriad of determinants emerged, which were reducing the duration of lectures, using mixed teaching techniques, making lectures more interactive, linking to clinical practice, having a flexible class schedule, delaying morning classes, and providing study breaks before exams. Linking lectures to clinical practice was the one that was most statistically significant (p=0.02), with males who expressed stronger agreement (59%). This depicts that medical students prefer that their lectures contain clinical elements that will help them solve real medical problems later. This point is further proven in a study by Sharma et al., which implied that “stressing the practical application of knowledge in classrooms was amongst the high-value factors seen to improve attendance” [21].

Another major factor that emerged was that of having a flexible class schedule. Students, mainly the exam-going ones (semester 6: 94.9%), strongly agreed that if they did not have to attend college six days a week, it would greatly help them in their attendance in class, as this leads to exhaustion and an inability for students to recover at their own pace, as demonstrated by Rehman et al. [19]. Delaying the early morning lectures was especially supported by students of Indian nationality (63.3%) as compared to a mean agreement rate of 31.2% among the other nationalities. A study by Saima et al. proved the same [22]. A study by Sengupta et al. found 88.7% of students recommended making lectures more interactive. A likewise pattern, a mean of 55.7% across the three semesters, was also noted in this study, strengthening that the use of mixed online and physical lectures would greatly improve attendance [23].

Factors Not Significantly Associated With Absenteeism

Health issues being a reason for absenteeism was only agreed upon by 28 out of 100 students (28%), showing that absenteeism was independent of sickness. However, the contrary was demonstrated by Garg, who showed that students, especially females, missed classes due to health reasons [24]. Less than half of the study population, 20 (20%) of students, strongly agreed that travel does not negatively affect their attendance. The same was reported in the study by Banerjee et al. [25].

Forty-three out of 100 students were neutral about the fact that lecture duration should be reduced to help improve attendance. However, the contrary was documented by Garg, whereby it was found that the general deterrent was the long duration of lectures, exceeding the 30-40 minute passive attention span” [24].

The idea of more interactive lectures was fully agreed upon by only 30 students, therefore showing that the students already found the pharmacology classes interactive enough. Having study breaks before the exam period was found to be a major factor in encouraging students to attend lectures. This is evident as the data collected had a mean of 50% of students agreeing to this. The same was found in Narayankar et al.'s study that the most common reason for missing class (42.2%) was "studying for exam; preparatory leave required before every exam," further reinforcing this argument [18].

Qualitative domain

Five themes emerged regarding the causes of absenteeism in pharmacology. Those themes were then broken down into categories, and the generated codes were assigned to them.

Peer Influence

As evidenced by Knifsend et al, students tend to be affected by their friends’ attendance. The interviewees agreed that they were more likely to skip classes if their friends were as well [26].

Attendance Pattern

The interviews revealed that some students tend to be regular, which makes up the category of consistency of attendance. However, in the same category, some admitted that they selectively attend classes whereby said classes are more interactive, i.e., students don’t want to attend lectures where they take notes. This is consistent with the results obtained by Gershenson, where the study stated that teachers modestly affect student attendance [27]. Under the same theme umbrella, attending pharmacology classes was said to establish a routine by interviewees despite the fact that Kiltie et al. found no overall relationship between routine (and routine change) and mental well-being of students; it might not affect mental well-being, but it seems to affect attendance. Students also reported attending classes for the simple purpose of gaining knowledge [28].

Academic and Teaching Influence

Two codes were classified under the category of teaching style: lack of engagement and proactive teachers. Another category pertaining to the same theme was learning methodology. According to Stoner et al., when pharmacy students are in the clinical application portions of their curriculum, they have reported that increased presentation and discussion of clinical scenarios is particularly beneficial. Similarly, some interviewees agree that they would attend classes more if the latter had more clinical application [29]. A third category was cognitive factors, and it included one of the most common causes of absenteeism that was also discussed in the quantitative domain: burnout. This is in line with our quantitative domain as well as the study conducted by Dabbagh et al., which concluded a high prevalence of depression, stress, burnout, and anxiety among medical trainees [30].

Institutional and Policy Factors

There is a fixed timetable that poses systemic limitations. Students don’t appreciate medical emergencies being counted as an absence, as the school has a percentage requirement of attendance to be able to appear for exams. However, the study conducted by Subramaniam et al. showed that increasing the percentage requirement of attendance resulted in students obtaining better grades [31].

Structural Barriers

Interviewees have reported that they are more likely to skip morning lectures. The quantitative domain also supported this view, as well as a study by Yeo et al., which showed that “attendance rates were about 10 percentage points lower in students taking classes at 08:00 compared with later class start times” [32].

Triangulation of quantitative and qualitative domains

Data from both the quantitative and qualitative domains showed strong convergence towards teaching-related factors influencing absenteeism in pharmacology. In the quantitative domain, the most statistically significant result (p=0.002) was that long, monotonous lectures accentuated absenteeism. These findings were consistent with the qualitative data, whereby students noted a lack of engagement/monotonous lectures (and students said, "They skip when content is dry”) under the academic and teaching influence theme. This convergence of the results was also found in the inflexible lecture schedules part, whereby quantitatively, with p=0.046, which is statistically significant for six consecutive classes a week, and qualitatively under the institutional and policy theme, where students identified the timetable as rigid and tiring. (Students want a “flexible schedule”). In addition to these, stress/burnout was found to be a major factor precipitating absenteeism, especially among exam-going students. This similar pattern also runs in parallel with the qualitative domain, precisely in the academic and teaching influence theme, where students cited “burnout” as a cognitive factor and “stress from heavy workload”.

Quantitatively, self-study was preferred by a large number of students (fifth semester, 33%). This was mirrored in the qualitative domain, namely in the academic and teaching theme, where students said they prefer self-directed study and attended the lecture session only if they thought it was worth attending. The theme of structural barriers also emerged in this study. Qualitatively, the “early morning lectures” were described as a “barrier” in the interviews. This finding was in line with the quantitative domain, where difficulty waking up greatly contributed to increasing absenteeism.

The aspect of incorporating clinical practice in lectures was firmly underscored in both domains. Quantitative data (p=0.02) showed a significant correlation that this factor helped to improve attendance, and in the qualitative part, students cited “clinical application valued highly” and were keen to attend sessions when the cases were being discussed. An overwhelming majority of students also strongly agreed that teachers must use interactive, mixed methods of teaching. Similar findings were reported by Robinson et al., who observed that the hybrid teaching model combines the strengths of didactic and online lectures, providing a balanced educational environment and improved learning for a wider range of students [33]. 

Quantitatively, 88% recommended the same, and qualitatively, students regarded “active lecture engagement” and “peer monitoring” as helping in attendance. Moreover, health issues and travel issues were not found to be significantly associated with absenteeism, both quantitatively and qualitatively.

Strengths of the study

The main strength of this study lies in its mixed-methods cross-sectional study design, which triangulates both qualitative and quantitative data to understand the causes and factors for improving attendance in pharmacology perceived by medical students. The integration of quantitative survey findings with qualitative thematic analysis strengthens interpretation by exploring not only the extent of absenteeism but also underlying reasons and perceptions among medical students. Inclusion of multiple academic cohorts' students from Semesters 4-6, allowing perspectives across different stages of pharmacology training. 

Limitations of the study

The limitation of this study is a moderate response rate of 69.44%, with 100 respondents participating in the study. Due to its cross-sectional design, this study does not allow causal conclusions to be drawn, and the findings are limited to observed associations. The study may be subject to self-report bias, including social desirability and recall bias, due to the self-reported nature of the data. Voluntary response bias may also have been present. In addition, non-response bias cannot be excluded, as non-respondents may have systematically different characteristics compared to respondents. However, anonymity and confidentiality were maintained to encourage honest responses. This study was conducted in a single medical college, which may limit the generalizability of the findings. A multicentric study in various medical colleges will represent a better representation of absenteeism across Mauritius.

## Conclusions

Absenteeism in pharmacology remains a significant and multifactorial issue supported by the quantitative and qualitative findings of this study. This study identifies several contributory factors underlying absenteeism, including long lectures (>1 hour), a preference for self-study, difficulty waking up, six consecutive classes per week, and burnout, which were perceived by students as influencing attendance.

The factors that may improve attendance were flexible class schedules, incorporating mixed teaching techniques, providing study breaks before examinations, reducing the duration of lectures, and delaying early morning classes (8:00 am). In addition, making lectures more interactive and linking content to clinical practice are essential and were perceived by students to improve attendance. 

## Appendices

Appendix A 

**Table 7:** Questionnaire

Section A: Demographic Details								
1	Gender		Female	Male				
2	Semesters		4th	5th	6^th^			
3	Age- years							
4	Nationality		Mauritian	Indian	South African		Other	
5	Current living arrangement		Hostel	Day scholar				
6	Commute to college		Walk	Bus	Car			
Section B: Factors causing absenteeism			1- strongly disagree	2- disagreed	3- neutral	4- agree		5- strongly agree
1	Skip lectures because of long lecture durations.		1	2	3	4		5
2	Skip classes as I can self study better .		1	2	3	4		5
3	Miss classes due to stress and burnout.		1	2	3	4		5
4	Difficult to wake up for early morning lectures.		1	2	3	4		5
5	Skip classes due to health issues.		1	2	3	4		5
6	Find it difficult to attend classes for six consecutive days a week.		1	2	3	4		5
7	Difficult to attend classes sometimes due to adverse weather.		1	2	3	4		5
8	Have travel issues.		1	2	3	4		5
Section C: Improvements			1- strongly disagree	2- disagreed	3- neutral	4- agree		5- strongly agree
1	Reducing the duration of lectures would help in improving attendance,		1	2	3	4		5
2	Use of mixed teaching techniques (online + physical classes) would be helpful.		1	2	3	4		5
3	Lectures should be made more interactive.		1	2	3	4		5
4	Link lectures to clinical practice to improve attendance.		1	2	3	4		5
5	The class schedule should be made more flexible.		1	2	3	4		5
6	Delay the early morning lectures.		1	2	3	4		5
7	To include study breaks before exams.		1	2	3	4		5
Section D: Qualitative Questionnaire								
1		How regular are you in pharmacology classes?						
2		What do you think about before, during and after missing a pharmacology lecture?						
3		Which types of classes or situations make you more likely to bunk?						
4		Do you feel that you are in control of your attendance? Why?						
5		How does burnout affect your attendance?						
6		How do your peers influence your attendance?						
7		What motivates you to attend classes?						
8		How do teachers/teaching methods affect your willingness to attend pharmacology classes?						
9		If attendance was not compulsory, would you still attend pharmacology classes? Why?						
10		What do you think would motivate you or even the other students to attend classes more regularly?						
